# Genetic analysis reveals conspecificity of two nominal species of *Anaphes* fairyflies (Hymenoptera: Mymaridae), egg parasitoids of *Oulema* leaf beetle (Coleoptera: Chrysomelidae) pests of cereal crops in Europe and of rice in East Asia

**DOI:** 10.1371/journal.pone.0273823

**Published:** 2023-01-27

**Authors:** S. V. Triapitsyn, P. F. Rugman-Jones, H. Kusuhara, R. Nakano, P. Janšta, S. Arikawa, T. Adachi-Hagimori

**Affiliations:** 1 Department of Entomology, University of California, Riverside, California, United States of America; 2 Laboratory of Insect Natural Enemies, Institute of Biological Control, Faculty of Agriculture, Kyushu University, Fukuoka, Japan; 3 Laboratory of Applied Entomology, University of Miyazaki, Miyazaki, Japan; 4 Shida-Haibara Agriculture and Forestry Office, Shizuoka, Japan; 5 Department of Entomology, State Museum of Natural History Stuttgart, Stuttgart, Germany; 6 Department of Zoology, Faculty of Science, Charles University, Prague, Czech Republic; Zhejiang University, CHINA

## Abstract

*Anaphes* (*Anaphes*) *flavipes* (Foerster), a fairyfly (Hymenoptera: Mymaridae) native of Europe, is an economically important egg parasitoid for the natural control of *Oulema* spp. leaf beetle (Coleoptera: Chrysomelidae) pests of cereal crops such as barley, oats, rye, and wheat in Europe, and for the classical biological control of the invasive *Oulema melanopus* (L.) in North America. A morphologically very similar *Anaphes* (*Anaphes*) *nipponicus* Kuwayama, known from mainland China, Japan, Republic of Korea, Far East of Russia and Taiwan, is an egg parasitoid of *Oulema oryzae* (Kuwayama), a pest of rice mainly in temperate parts of East Asia. The nuclear 28S-D2 and ITS2 and the mitochondrial COI genes were used as markers to compare specimens of *A*. (*Anaphes*) *flavipes* reared from eggs of an *Oulema* sp. on barley in Germany with those of *A*. (*Anaphes*) *nipponicus* reared from eggs of *O*. *oryzae* on rice in Honshu Island, Japan. Because the resulting sequences are practically identical, within an expected intraspecific genetic variability, conspecificity of these two nominal species has been confirmed, and consequently *A*. (*Anaphes*) *nipponicus* Kuwayama, 1932, syn. n. is synonymized with *A*. (*Anaphes*) *flavipes* (Foerster, 1841). Taxonomic notes and illustrations are provided for the specimens of both sexes of *A*. (*Anaphes*) *flavipes* from Japan to facilitate their recognition.

## Introduction

**Fig 1:**
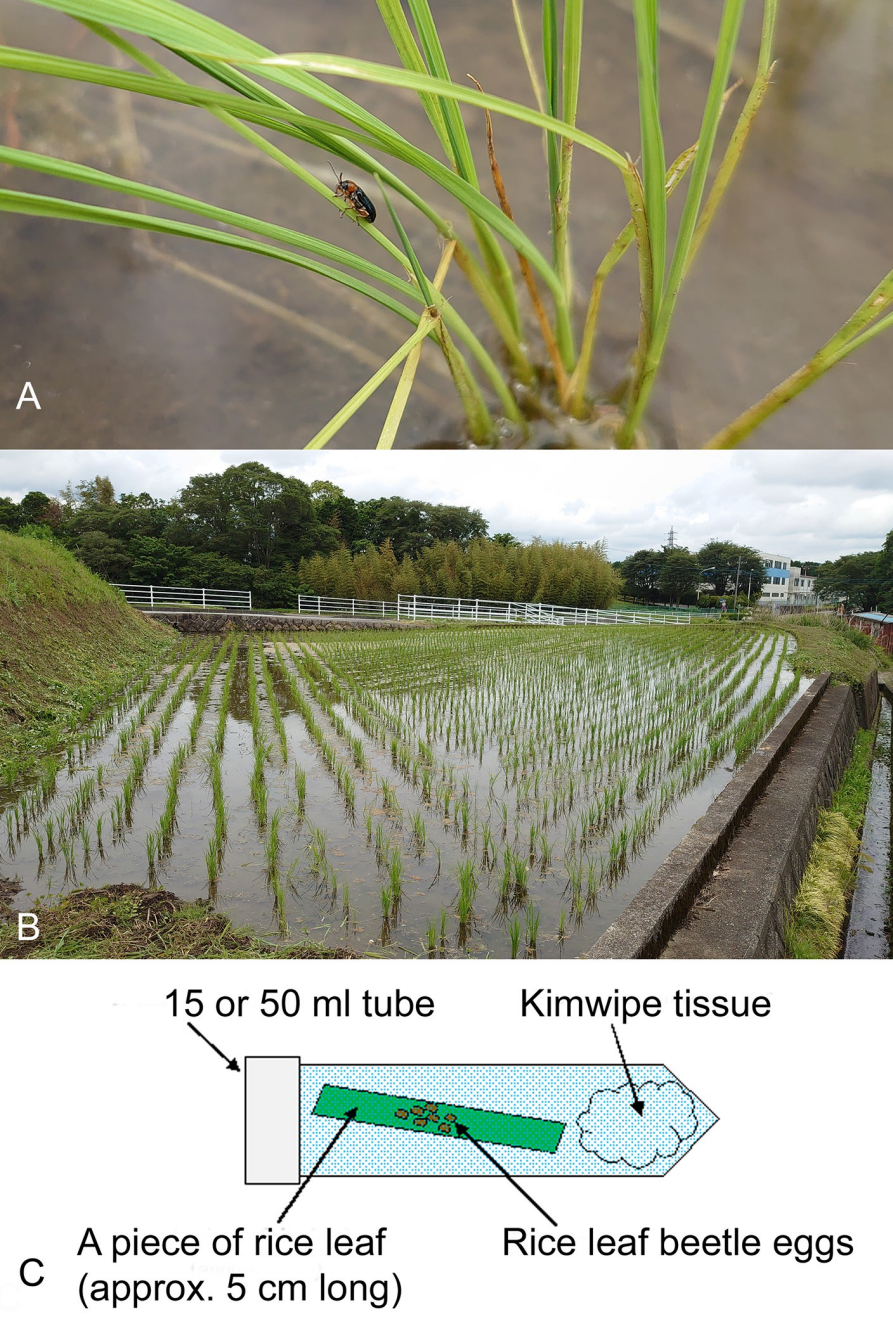
A) *Oulema oryzae* rice leaf beetle on a rice plant in Oyama, Shizuoka Prefecture, Japan; 2) one of the sampled paddy fields in Oyama (35°20’51”N 138°58’02”E); C) rearing method of egg parasitoids of *O*. *oryzae* from Oyama.

Leaf beetles of the genus *Oulema* Des Gozis (Coleoptera: Chrysomelidae) are rather common pests, mainly in the Palaearctic region, of cereal crops such as barley, oats, rye, and wheat in Europe as well as rice in East Asia. *Oulema melanopus* (L.), one of the several species of the genus native to Europe, was unintentionally introduced to North America where it became a serious pest of wheat and other cereals [1–3]. A classical biological control program was implemented in North America against the invasive *O*. *melanopus* resulting in the deliberate introduction of the European fairyfly *Anaphes* (*Anaphes*) *flavipes* (Foerster) (Hymenoptera: Mymaridae), in natural conditions a known egg parasitoid of *Lema* Fabricius and *Oulema* species [4]. As the result, *A*. *flavipes* is now well established in Canada and the USA where it is considered to be an important biological control agent. Additionally, attempts of a neoclassical biological control were made by evaluating, under quarantine laboratory conditions in Washington State, USA, *Anaphes nipponicus* Kuwayama from Fujian, China [5, 6], which readily attacked, oviposited and successfully completed two generations on eggs of *O*. *melanopus*; however, due to inability to develop successfully at low humidity it was not considered to be a promising biocontrol agent in eastern Washington [6]. *Anaphes nipponicus* was originally described from Hokkaido Island, Japan, as an egg parasitoid of the rice leaf beetle *Oulema oryzae* (Kuwayama) (Fig 1A), a pest of rice mainly in the temperate zones of East Asia, particularly in its Palaearctic parts. This parasitoid species was originally described from a syntype series from the following two type localities: Ōno in Kameda District (now Hokuto in Oshima Subprefecture of Hokkaido Prefecture), and Kagura, Kamikawa District (now Higashikagura in Kamikawa Subprefecture of Hokkaido Prefecture) [7]. The coordinates of these type localities, as indicated by Huber & Thuróczy (2018) [8], seem to be incorrect, particularly of the Ōno site which are not on land. Besides Fujian, China, which is within the Oriental part of the Palaearctic region, *A*. *nipponicus* was also reared from eggs of *O*. *oryzae* in Primorskiy Kray of Russia [9–11].

Ironically, until recently the taxonomic identities of both *A*. *flavipes* and *A*. *nipponicus* were unclear, and the former nominal species in particular was often misidentified unless being reared from eggs of *Lema* or *Oulema* hosts on cereals [1, 2]. For *A*. *flavipes* at least, that was mitigated by Samková *et al*. (2017) [2] when the species was thoroughly redescribed and illustrated, so it became more or less recognizable, considering difficulties of identifying *Anaphes* Haliday species in general. It was very important that the redescription included a thorough morphometric analysis indicating wide ranges for the key diagnostic features. At the same time, even though *A*. *nipponicus* was also partially redescribed and illustrated in Samková *et al*. (2017) [2] and Triapitsyn (2021) [12], the lack of well-prepared, freshly collected, reared material from the known host of this species, recognizing it remained a taxonomic problem because morphologically they are very similar. Although Samková *et al*. (2017) [2] indicated some very minor differences between these two nominal species of *Anaphes*, Triapitsyn (2021) [12] found them to be either within or very close to the known morphological variability ranges of *A*. *flavipes*, so in his key to the Palearctic species of the nominate subgenus of *Anaphes*, to which they belong, they were separated only by their geographical distribution and the host associations. It was also indicated that a genetic comparison of these two nominal species would be very desirable to confirm their possible conspecificity [12]. Without supporting molecular data, it was impossible to make a well-justified decision regarding their taxonomic status because of these notable differences in the distributional ranges, habitats and host associations, even though they attacked eggs of the same leaf beetle genus (*Oulema*). Here, we present and analyze such genetic data for both nominal species based on reared specimens that reveal their conspecificity.

## Materials and methods

### Collection of samples

In Germany, specimens of *A*. *flavipes* were reared from eggs of *Oulema* sp. on barley in Aachen and its environs, North Rhine-Westphalia, in the general area of both the original type locality(es) of this parasitoid and also at and near the type locality of its neotype designated by Samková *et al*. (2017) [2]. Because we used the ethanol-preserved (stored in a freezer since June 2011) voucher specimens of their study, including a few from the same reared series as the neotype, the collecting methods described in Samková *et al*. (2017) [2] fully apply to our specimens from Germany as well and thus do not need to be repeated.

**Fig 2:**
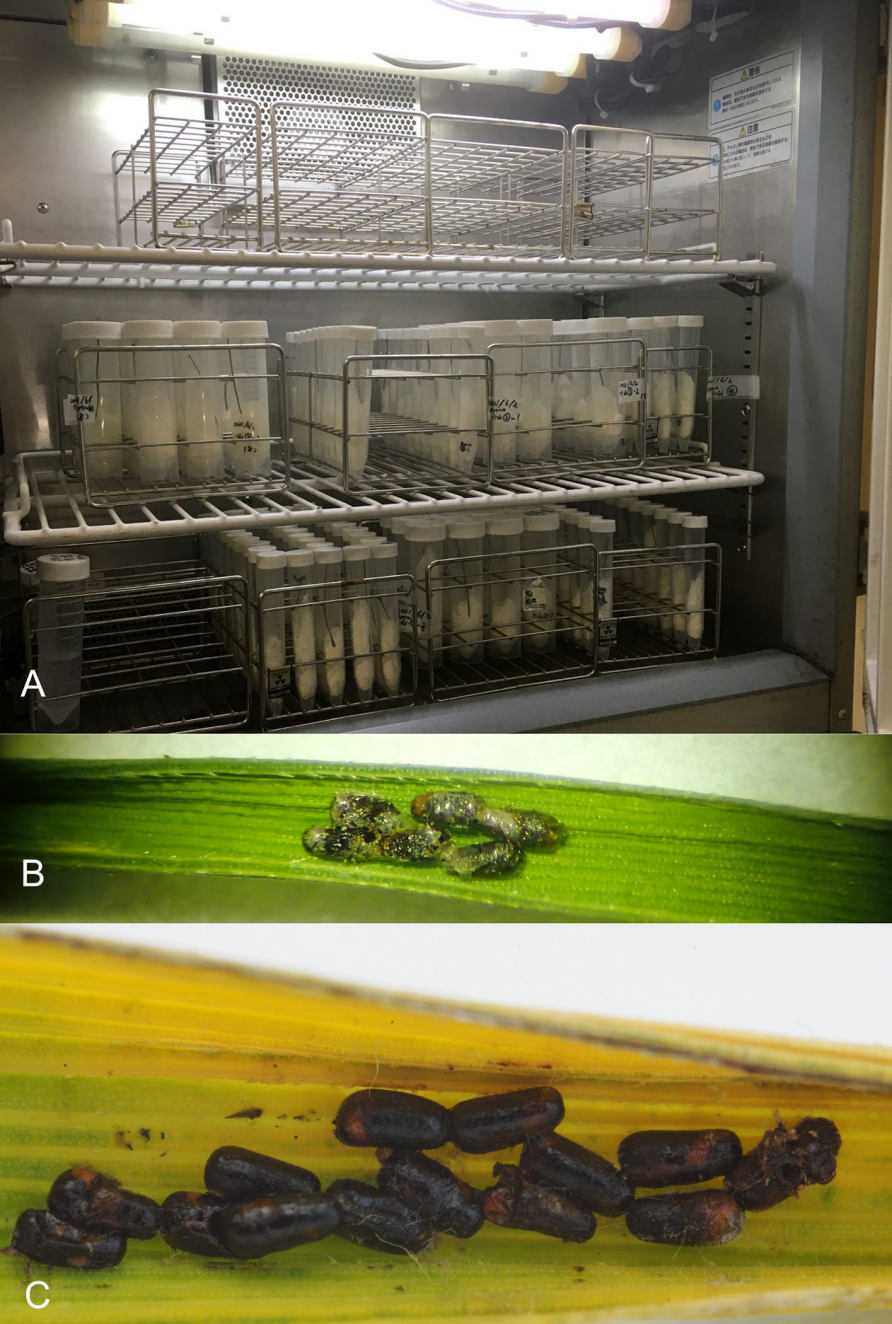
A) An incubator used to rear egg parasitoids of *Oulema oryzae* from Oyama, Shizuoka Prefecture, Japan; B) pupae of *Anaphes flavipes* from Oyama; C) eggs of *O*. *oryzae* from Koriyama, Fukushima Prefecture, Japan parasitized by *Anaphes flavipes* (after emergence).

**Fig 3:**
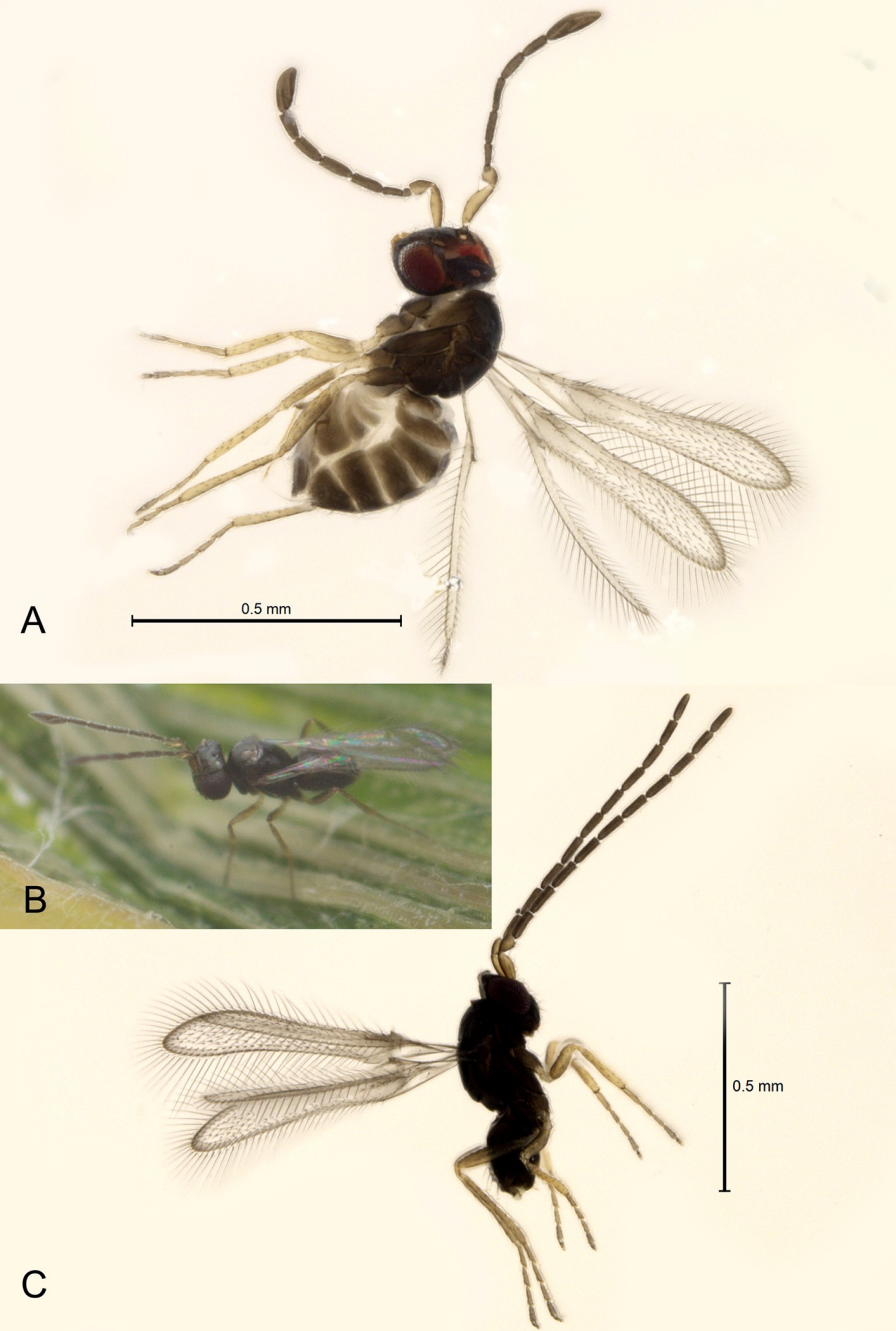
*Anaphes flavipes* from Japan: A) female from Koriyama, Fukushima Prefecture (in ethanol); B) female from Koriyama (alive); C) male from Oyama, Shizuoka Prefecture (in ethanol).

In Japan, rearing of *A*. *nipponicus* from eggs of *Oulema oryzae* in paddy fields was conducted in two different locations on Honshu Island. In Shizuoka Prefecture, egg masses of *O*. *oryzae* were collected 2.June.2021 by R. Nakano on rice plants in 4 non-organic paddy fields located within a 250 m radius (Table 1; Fig 1B) in Oyama, Sunto District. Each of the approximately 5 cm long pieces of rice leaves with *O*. *oryzae* eggs was placed in a 15 ml or 50 ml plastic centrifuge tube together with a crumpled piece of Kimwipes® tissue (Fig 1C), and placed in an incubator at 20°C, 50% relative humidity, and constant light from 7:00 to 23:00 in the laboratory (Fig 2A). The samples were checked daily for parasitoid emergence. The reared adult *A*. *nipponicus* were collected by T. Adachi-Hagimori from a few parasitized eggs of *O*. *oryzae* (Fig 2B); 1 female and 2 males (Fig 3C) emerged 9–13. June.2021 and were stored in 95% ethanol in a freezer at -20°C until shipped to S. V. Triapitsyn and P. F. Rugman-Jones for morphological and molecular identification, respectively.

**Table 1:** Summary of the collections of *Oulema oryzae* egg masses in Oyama, Sunto District, Shizuoka Prefecture, Japan on 2.vi.2021.

Rice field number	GPS coordinates of location	Number of *O*. *oryzae* egg masses
Oyama_1	35°20’51.3"N 138°58’04.3"E	49
Oyama_2	35°20’51.6"N 138°58’08.9"E	39
Oyama_3	35°20’51.0"N 138°58’01.9"E	35
Oyama_4	35°20’48.3"N 138°58’18.6"E	21
		Total: 144

**Fig 4:**
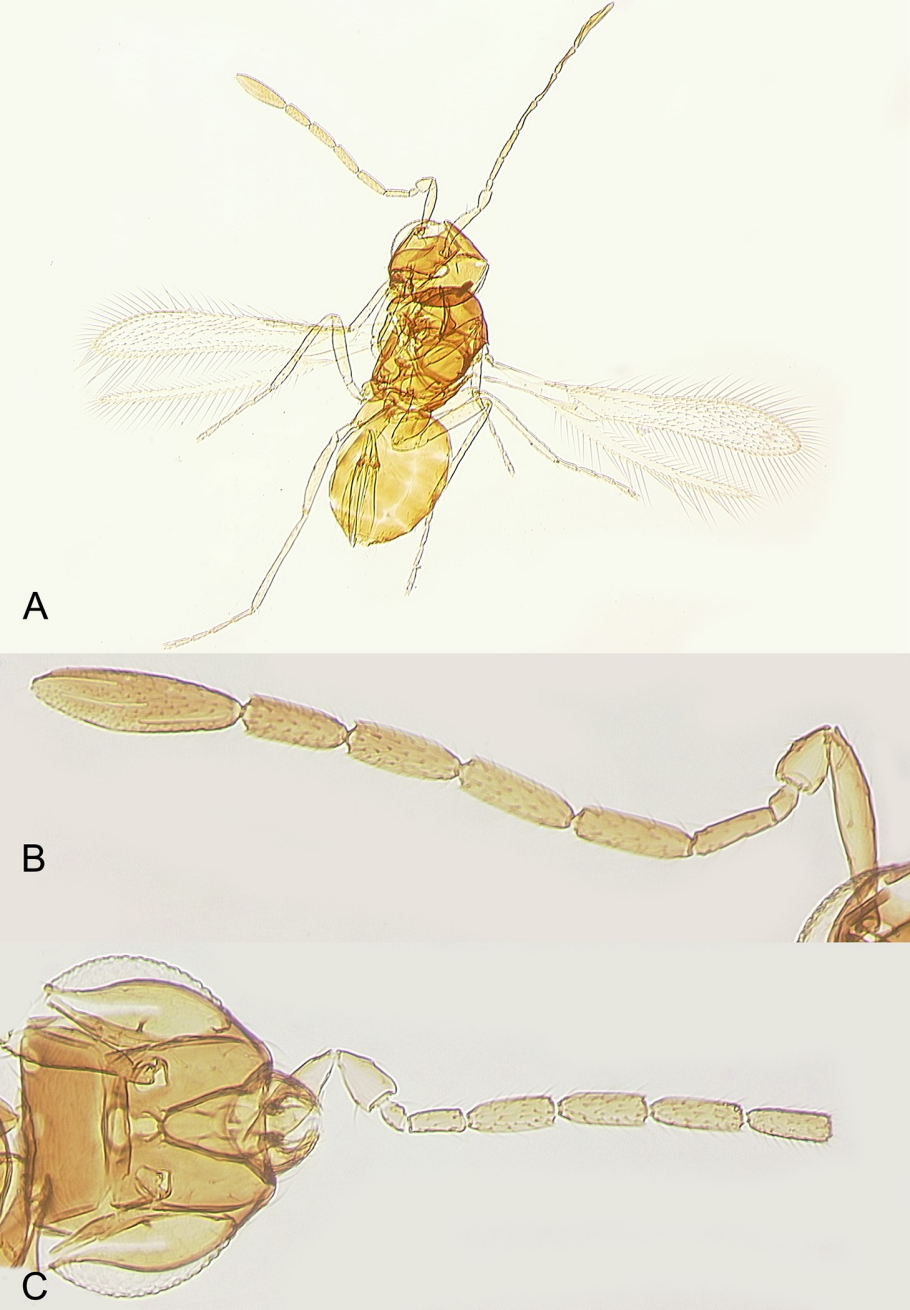
*Anaphes flavipes* females from Koriyama, Fukushima Prefecture, Japan: A) habitus; B) antenna of a larger specimen with F2 relatively long; B) head and antenna of a smaller specimen with F2 relatively short (clava is missing).

In Fukushima Prefecture, egg masses of *O*. *oryzae* were collected 7.July.2021 by S. Arikawa on rice plant leaves in an organic field in Mihota-machi, Nabeyama, Koriyama City (37°20’37”N 140°17’45”E, 263 m). The samples were checked daily in the laboratory for emergence, and the reared adult *A*. *nipponicus* were collected by H. Kusuhara from the parasitized eggs of *O*. *oryzae* (Fig 2C); 8 females (Figs 3A, 3B and 4A) and 3 males emerged 12-13.July.2021 and were stored in 99.5% ethanol in a freezer at -20°C until shipped to S. V. Triapitsyn and P. F. Rugman-Jones for morphological and molecular identification, respectively.

### Taxonomic studies

For morphological terminology we follow that of Triapitsyn (2021) [12]. An abbreviation for a funicular segment of female antenna is F.

Molecular voucher specimens of *A*. *flavipes* were slide-mounted in Canada balsam using a slightly modified technique described in Huber (2015) [13]. All slide mounts were examined under a Zeiss Axioskop 2 plus compound microscope (Carl Zeiss Microscopy, LLC, Thornwood, New York, USA).

The following acronyms are used to designate depositories of voucher and other specimens examined:

ELKU Entomological Laboratory, Faculty of Agriculture, Kyushu University, Fukuoka, Japan.
UCRC Entomology Research Museum, University of California, Riverside, California, USA.

### DNA extraction, amplification, sequencing, and genetic data analysis

Genomic DNA was extracted from nine individual wasps (4 *A*. *flavipes* from Germany and 5 *A*. *nipponicus* from Japan) using the non-destructive HotSHOT method of Truett *et al*. (2000) [14] in a total volume of 50 μL. Following DNA extraction, all specimens were retrieved and individually slide-mounted for morphological examination (see above). Each molecular voucher specimen was assigned a P. F. Rugman-Jones’ molecular voucher PR number and UCRC database unique identifier number. Extracted DNA was stored at -20°C. Three separate loci were amplified using the polymerase chain reaction: the 5’ region of mitochondrial cytochrome c oxidase subunit I (COI) using the LCO1490 and HCO2198 primer set as described in Triapitsyn *et al*. (2021) [15]; and, the internal transcribed spacer 2 (ITS2) and D2 region of 28S (28S-D2) of ribosomal RNA using respective primer sets and protocols described in Morse *et al*. (2016) [16]. Amplifications were confirmed by gel electrophoresis, purified, and direct sequenced in both directions at the Institute for Integrative Genome Biology, University of California at Riverside, California, USA. The parity of forward and reverse reads was checked using SEQUENCHER 4.9 (Gene Codes Corporation, Ann Arbor, MI, USA) and priming regions were removed manually in BioEdit version 7.0.5.3 [17]. The online tool EMBOSS Transeq [18] was used to translate the protein coding COI sequences into their amino acid chains, confirming the absence of indels and pseudogenes in the final dataset. Short flanking sequences of 5.8S and 28S were identified, and subsequently removed, using the “annotate” function in the online ITS2 database [19, 20]. Given the limited aims of our study, genetic analyses were restricted to simple comparison of DNA sequences of each locus among the sequenced specimens, and with those held in two public repositories, GenBank [21] and BOLD [22]. Sequences of each locus were aligned in MAFFT online using the Q-INS-I strategy. All sequences generated herein were deposited in GenBank.

## Results

### Sequence analysis

We obtained 28S-D2 and ITS2 sequences from all 9 specimens, and a COI sequences from 8 of the 9 specimens. The sequences generated from the 4 individuals of *A*. *flavipes* from Germany were found to be almost identical to those of 5 individuals of *A*. *nipponicus* from Japan. Sequences of 28S-D2 were identical in length (520 bp) and nucleotide composition across all 9 specimens (GenBank accessions OM701783-701791) with the exception that a single German specimen (PR21-489) was polymorphic at a single position (S1 Table). Sequences of the typically much more variable ITS2 were also very similar in both length (514–517 bp) and composition with variation limited to a handful of single base substitutions or the insertion/deletion of a microsatellite repeat motif (OM701774-701782; S2 Table). No significant matches (>97%) were found in GenBank for 28S-D2 or ITS2. Sequences of the COI of the five specimens of *A*. *nipponicus* were identical (OM687258-687262) and these differed from those of three *A*. *flavipes* (OM687255-687257) specimens at only 3 positions (each a synonymous substitution; positions 148, 319, and 337; S3 Table). One of those positions (319) was also variable within *A*. *flavipes*, but at a maximum of <0.5%; variation among the sequences was well within that expected at the intraspecific level. Taken together, sequences of these three loci provide conclusive evidence that the specimens of *A*. *flavipes* and *A*. *nipponicus* represent a single species. Comparison of the COI sequences with BOLD revealed the existence of several “private” accessions (identified only to genus) originating from specimens apparently collected as far and wide as Canada, Germany, Bangladesh, and Vietnam.

### Taxonomy

#### *Anaphes* (*Anaphes*) *flavipes* (Foerster, 1841)

Figs 3–5

**Fig 5:**
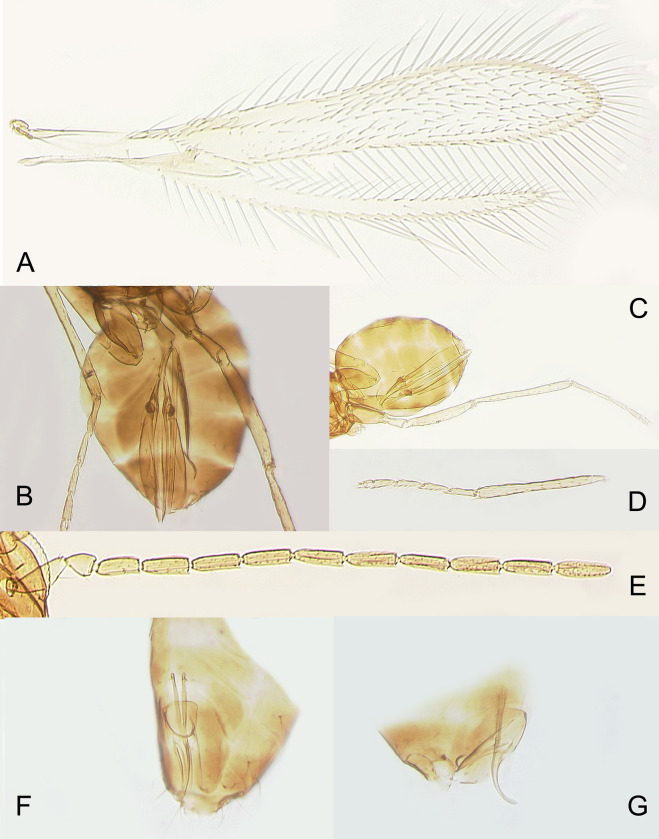
*Anaphes flavipes* from Japan: A) female fore and hind wings (Koriyama, Fukushima Prefecture); B) female gaster (Oyama, Shizuoka Prefecture); C) female gaster and hind leg (Koriyama); D) female metatibia and metatarsus (Koriyama); E) male antenna (Koriyama); F) male genitalia in dorsal view (Oyama); G) male genitalia in lateral view (Koriyama).

*Gonatocerus flavipes* Foerster, 1841 [23]: 45.

*Anaphes nipponicus* Kuwayama, 1932 [7]: 93. **Syn. n.**

*Anaphes nipponicus* Kuwayama: Togashi, 1974 [24]: 12 (host egg parasitism); Huber, 1992 [1]: 75 (list); Storozheva, 1989 [9]: 14–16 (host in the Russian Far East); Storozheva, 1990a [10]: 113 (host); Storozheva, 1990b [11]: 29 (parasitism, biology); Samková *et al*., 2017 [2]: 690–697 (taxonomic history, type information, redescription, comparison with *A*. *flavipes*, distribution, host association).

*Anaphes flavipes* (Foerster) [or as Förster]: Huber, 1992 [1]: 36 (key), 47–50 (taxonomic history, synonyms, type information, redescription, distribution, hosts, discussion), 74 (list), 100, 104 (illustrations); Samková *et al*., 2017 [2]: 677–690 (neotype designation, taxonomic history, synonymy, redescription, comparison with *A*. *nipponicus*, distribution, host associations).

*Anaphes* (*Anaphes*) *flavipes* (Foerster): Huber & Thuróczy, 2018 [8]: 25–26 (list, type information, synonyms), 45 (key), 88 (illustration); Huber *et al*., 2020 [25]: 68–69 (taxonomic history, list of synonyms, hosts and distribution in the Nearctic region); Triapitsyn, 2021 [12]: 7 (key), 17–21 (taxonomic history, distribution, diagnosis, hosts, discussion, illustrations).

*Anaphes* (*Anaphes*) *nipponicus* Kuwayama: Huber & Thuróczy, 2018 [8]: 27 (list, type information); Triapitsyn, 2021 [12]: 7 (key), 33–36 (taxonomic history, distribution, diagnosis, host, discussion, illustrations).

MATERIAL EXAMINED. **Germany:** North Rhine-Westphalia: Aachen, 50.77876°N 6.03816°E, 198 m, 3.June.2011, P. Janšta (emerged from eggs of *Oulema* sp. on barley) [3 ♀**♀**, 3 ♂**♂**, UCRC]; Am Gericht (near Simmerath, 25 km SE of Aachen), 50.59192°N 6.28775°E, 555 m, 2.June.2011, P. Janšta, A. Samková (emerged from eggs of *Oulema* sp. on barley) [3 ♀♀, 3 ♂♂, UCRC (including molecular vouchers: 1 ♀, PR21-490, UCRC_ENT 00541260 and 1 ♂, PR21-491, UCRC_ENT 00541261)]; near Merken, 50.85732°N 6.40682°E, 103 m, 1.June.2011, P. Janšta (emerged from eggs of *Oulema* sp. on barley) [3 ♀**♀**, 3 ♂, UCRC]; Uersfeld (5 km N of Aachen), 50.82178°N 6.06889°E, 174 m, 2.June.2011, P. Janšta, A. Samková (emerged from eggs of *Oulema* sp. on barley) [3 ♀♀, 3 ♂♂, UCRC (including molecular vouchers: 1 ♀, PR21-488, UCRC_ENT 00541258 and 1 ♂, PR21-489, UCRC_ENT 00541259)]. **Japan:** Honshu Island: Fukushima Prefecture, Koriyama City, Nabeyama, Mihota-machi, 37°20’37”N 140°17’45”E, 263 m (emerged 12-13.July.2021, reared by H. Kusuhara from parasitized eggs of *Oulema oryzae* collected 7.July.2021 by S. Arikawa in organic rice field) [8 ♀♀, 3 ♂♂, ELKU, UCRC (including molecular vouchers: 1 ♀, emerged 12.July.2021, PR21-494, UCRC_ENT 00541264 as well as 1 ♀, emerged 13.July.2021, PR21-496, UCRC_ENT 00541266 and 1 ♂, emerged 13.July.2021, PR21-495, UCRC_ENT 00541265)]. Ishikawa Prefecture, Wajima, July.1973, I. Togashi, from eggs of *O*. *oryzae* on rice [5 ♀♀, 3 ♂♂, ELKU] (determined by T. Tachikawa in 1976). Shizuoka Prefecture, Sunto District, Oyama (reared by T. Adachi-Hagimori from parasitized eggs of *O*. *oryzae* collected 2.June.2021 by R. Nakano in non-organic rice fields): 35°20’51”N 138°58’02”E, 401 m [2 ♂♂ (emerged 9.June.2021), UCRC, including molecular voucher PR21-493, UCRC_ENT 00541263]; 35°20’52”N 138°58’09”E, 397 m [1 ♀ (emerged 13.June.2021), UCRC, molecular voucher PR21-492, UCRC_ENT 00541262].

DIAGNOSIS. FEMALE. Diagnosed, redescribed and illustrated in detail by Samková *et al*. (2017) [2] based on specimens from Germany, from which a lectotype was designated. Triapitsyn (2021) [12] provided diagnoses and illustrations of both *A*. *flavipes* (based on specimens from Europe) and *A*. *nipponicus* (based on specimens from Wajima, Ishikawa Prefecture, Japan). Here we provide illustrations of the habitus (Figs 3A, 3B and 4A), antenna (Fig 4B and 4C), fore and hind wings (Fig 5A), metatarsus (Fig 5C and 5D) and ovipositor (Fig 5B and 5C) of the newly collected, good quality reared specimens from Japan to facilitate its recognition. In these slide-mounted specimens, body length 0.6–0.72 mm; antenna (Fig 4B and 4C) with F2 length very variable (sometimes very short in smaller specimens, Fig 4C), 2.4–4.0× as long as wide, and the combined length of F1 and F2 from slightly shorter than F3 to about as long as or slightly longer than F3, funicle with multiporous plate sensilla only on F3–F6 (2 on each), clava 3.5–3.6× as long as wide, a little shorter (0.9–0.95×) than the combined length of F5 and F6, with 6 multiporous plate sensilla; fore wing (Fig 5A) 0.57–0.72 mm long, 6.9–7.0× as long as wide, longest marginal seta 1.3–1.4× maximum wing width, marginal space separated from medial space by 1 complete line of setae; metatarsomere 1 at most about as long as metatarsomere 2 (Fig 5C and 5D); ovipositor occupying 0.75–0.8× length of gaster (Fig 5B and 5C), not exserted beyond its apex, and about 1.1× as long as metatibia.

MALE. Redescribed by Samková *et al*. (2017) [2] for both *A*. *flavipes* and *A*. *nipponicus*. Here we provide illustrations of the habitus (Fig 3C), antenna (Fig 5E), and genitalia (Fig 5F and 5G) of the specimens from Japan.

DISTRIBUTION. Austria, Bulgaria, Czech Republic, France, Germany, Ireland, Italy, Netherlands, Poland, Romania, Russia (also as *A*. *nipponicus* in the Russian Far East), Serbia, Ukraine, United Kingdom [12]; some other records [26] need verification; also known, as *A*. *nipponicus*, from China (Fujian, Taiwan), Japan, and Republic of Korea [1, 2, 5, 8, 12]. Introduced and established in Canada and USA [1, 2, 4].

HOSTS. Chrysomelidae: *Lema* spp. and *Oulema* spp. including *O*. *duftschmidi* (Redtenbacher), *O*. *gallaeciana* (Heyden), *O*. *melanopus* (L.), and *O*. *oryzae* (Kuwayama) [1–3, 12, 25, 27] (also as *A*. *nipponicus*).

Various aspects of biology, ecology and parasitism of *A*. *flavipes* in Europe were studied by Donev (1987) [27] and Samková *et al*. (2019a, 2019b, 2019c, 2020, 2021) [3, 28–31].

REMARKS. Triapitsyn (2021) [12] looked for the missing syntypes of *A*. *nipponicus* in the collection of Insect Museum, National Institute for Agro-Environmental Sciences, NARO, Tsukuba, Ibaraki, Japan (ITLJ), to where S. Kuwayama’s collection had been moved [2], during a brief visit in November 2019, but could not locate any. These appear to be lost. However, conspecificity of our specimens from Honshu Island with Kuwayama’s *A*. *nipponicus* from the nearby Hokkaido Island is not in doubt as they were reared from eggs of the same beetle host in a similar habitat of a paddy field, and are identical to the other known specimens of this species [2, 12, 24].

## Discussion

Morphologically, the newly reared specimens of *A*. *nipponicus* from Japan largely fit the known ranges of the diagnostically important features of both *A*. *flavipes* from Europe and *A*. *nipponicus* from Japan, as indicated in Samková *et al*. (2017) [2] and Triapitsyn (2021) [12]. Genetically, our analysis of the sequences of the selected mitochondrial and nuclear ribosomal gene regions unambiguously showed that these two nominal species are practically identical, well within an expected intraspecific genetic variability. Thus, their conspecificity is not in doubt, hence the synonymy of *A*. *nipponicus* under the much earlier described *A*. *flavipes*. Application of molecular methods for insect diagnostics has made it possible to resolve almost a century old misidentifications of these economically important natural enemies of key cereal agricultural crops.

In this particular case, identity of the *A*. *flavipes* individuals from Europe from which DNA was extracted, and then selected mitochondrial and nuclear ribosomal gene regions were sequenced, was not in doubt because these came from the same rearings and collections near Aachen, Germany, as the neotype (the ultimate identity defining specimen mounted on a slide) and other specimens on which morphological redescription of this species was based [2]. Therefore, this unfunded investigation was focused solely on revealing the true identity of *A*. *nipponicus* from Japan, which was not clear before this study, rather than on determining genetic variability of *A*. *flavipes* in Europe, that by itself warrants a separate study which would require substantial funding and effort to rear this egg parasitoid from different hosts throughout its range.

Although this conclusion about conspecificity of *A*. *flavipes* and *A*. *nipponicus* might be somewhat surprising considering their different geographical distribution, habitats and host associations (albeit parasitizing different species in the same host genus, *Oulema*), as well as obvious ecological differences of the respective agroecosystems between the cereal crops in Europe and paddy fields in temperate East Asia, similar examples do occur in Mymaridae. For instance, *Anagrus incarnatus* Haliday from Europe was found, using similar molecular methods, to be conspecific with *A*. *nilaparvatae* Pang & Wang from Asia, a well-known egg parasitoid of rice leafhoppers and planthoppers (Hemiptera: Cicadellidae and Delphacidae, respectively) [32].

Based on our collection data, parasitism of *O*. *oryzae* eggs on rice plants in the two sampled localities on Honshu Island in Japan seemed to be very low: for instance, out of the total 144 egg masses (individual eggs were not counted) of the leaf beetle host in the four paddy fields in Oyama, Shizuoka Prefecture, each of which contained several eggs, only one female and 2 male adult *A*. *flavipes* wasps emerged from one egg mass (Fig 2B) from the Oyama_3 field (Fig 1B; Table 1). Thus, the actual parasitism rate of *O*. *oryzae* eggs by *A*. *flavipes* in these sampled rice fields was less than 1%. That, however, might be due to the fact that these were non-organic, conventional rice fields with a history of prior pesticide use. Togashi (1974) [24], however, indicated 1.8–38.3% egg parasitism of *O*. *oryzae* in Ishikawa Prefecture, on the same island, mentioning that the highest percent parasitism at Tsurugi-machi (37.5%) and one area of Wajima City (38.3%) could have been due to preservation of the paddy fields in ancestral condition with more ecological diversity, thus probably providing a better habitat for the overwintering parasitoids.

## Supporting information

S1 Table DNA sequence alignment of a 520bp fragment of 28S-D2 rRNA from the nominal species *Anaphes flavipes* from Germany (PR21-488 thru 491) and *A*. *nipponicus* from Japan (PR21-492 thru 496). Shaded regions indicate nucleotide polymorphism.
(DOCX)

S2 Table Alignment of ITS2 rRNA sequences from the nominal species *Anaphes flavipes* from Germany (PR21-488 thru 491) and *A*. *nipponicus* from Japan (PR21-492 thru 496). Sequences were aligned in MAFFT and alignment gaps are designated using “-“. Shaded regions indicate areas containing nucleotide differences.
(DOCX)

S3 Table DNA sequence alignment of a 650bp fragment of the mitochondrial COI from the nominal species *Anaphes flavipes* from Germany (PR21-488 thru 491) and *A*. *nipponicus* from Japan (PR21-492 thru 496). Shaded regions indicate nucleotide differences.
(DOCX)
