# Supplementary material for: Genetic analysis reveals conspecificity of two nominal species of Anaphes fairyflies (Hymenoptera: Mymaridae), egg parasitoids of Oulema leaf beetle (Coleoptera: Chrysomelidae) pests of cereal crops in Europe and of rice in East Asia
Source: PLoS One. 2023 Jan 27;18(1):e0273823. doi: 10.1371/journal.pone.0273823 (PMC9882753; doi:10.1371/journal.pone.0273823)
Supplement: S2 Table — Sequences were aligned in MAFFT and alignment gaps are designated using “-“. Shaded regions indicate areas containing nucleotide differences. (DOCX) [file pone.0273823.s002.docx]

**S2 Table.** Alignment of ITS2 rRNA sequences from the nominal species *Anaphes flavipes* from Germany (PR21-488 thru 491) and *A. nipponicus* from Japan (PR21-492 thru 496). Sequences were aligned in MAFFT and alignment gaps are designated using “-“. Shaded regions indicate areas containing nucleotide differences.
